# Comparative Analysis of Conventional and Emerging Technologies for Seawater Desalination: Northern Chile as A Case Study

**DOI:** 10.3390/membranes11030180

**Published:** 2021-03-05

**Authors:** Aldo Saavedra, Hugo Valdés, Andrea Mahn, Orlando Acosta

**Affiliations:** 1 Departamento de Ingeniería Química, Facultad de Ingeniería, Universidad de Santiago de Chile (USACH), Av. Libertador Bernardo O’Higgins 3363, Estación Central 9160000, Chile; aldo.saavedra@usach.cl (A.S.); andrea.mahn@usach.cl (A.M.); 2 Centro de Innovación en Ingeniería Aplicada (CIIA), Departamento de Computación e Industrias, Facultad de Ciencias de la Ingeniería, Universidad Católica del Maule (UCM), Av. San Miguel 3605, Talca 3460000, Chile; 3 Gestionare Consultores, Carlos Antunez 2025 of. 608, Providencia 7500000, Chile; oacosta@gestionare.cl

**Keywords:** seawater desalination, emerging technologies, conventional technologies, thermal technologies, membrane technologies

## Abstract

The aim of this work was to study different desalination technologies as alternatives to conventional reverse osmosis (RO) through a systematic literature review. An expert panel evaluated thermal and membrane processes considering their possible implementation at a pilot plant scale (100 m^3^/d of purified water) starting from seawater at 20 °C with an average salinity of 34,000 ppm. The desalination plant would be located in the Atacama Region (Chile), where the high solar radiation level justifies an off-grid installation using photovoltaic panels. We classified the collected information about conventional and emerging technologies for seawater desalination, and then an expert panel evaluated these technologies considering five categories: (1) technical characteristics, (2) scale-up potential, (3) temperature effect, (4) electrical supply options, and (5) economic viability. Further, the potential inclusion of graphene oxide and aquaporin-based biomimetic membranes in the desalinization processes was analyzed. The comparative analysis lets us conclude that nanomembranes represent a technically and economically competitive alternative versus RO membranes. Therefore, a profitable desalination process should consider nanomembranes, use of an energy recovery system, and mixed energy supply (non-conventional renewable energy + electrical network). This document presents an up-to-date overview of the impact of emerging technologies on desalinated quality water, process costs, productivity, renewable energy use, and separation efficiency.

## 1. Introduction

Desalination is a separation process intended to increase water availability in structurally water-deficient countries that suffer recurrent periods of drought. Recently, the International Desalination Association (IDA) [1] reported that 150 countries apply desalination, based on daily activities of more than 300 million people worldwide. Between 2016 and 2019, the number of desalination plants and the daily water production increased by 12.4% and 41.2%, respectively, proving the accelerated growth of this technology [1,2].

**Figure 1:**
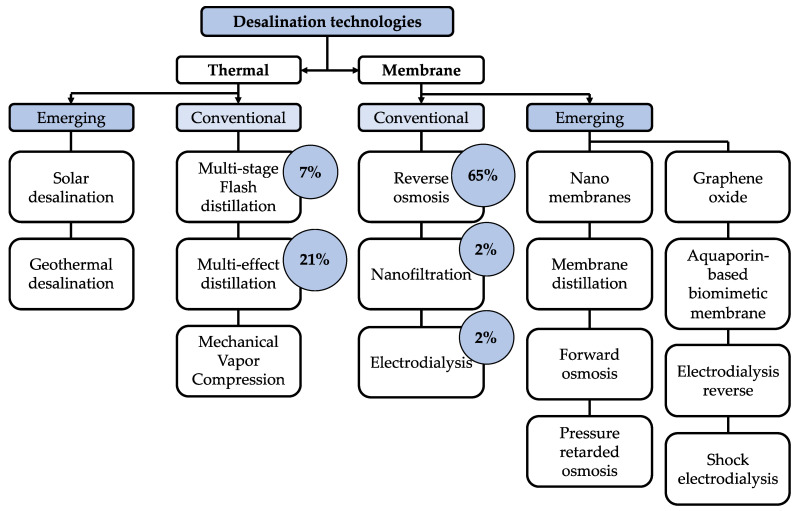
Schematic classification of desalination technologies.

Saudi Arabia has the largest water-production installed capacity, with 12 Mm^3^/d, representing 9.81% of the worldwide capacity, followed by the United Arab Emirates, the United States of America, Spain, and China, at 7.5, 4.7, 3.6, and 3.0%, respectively. Installation of desalination plants is mostly preferred when there is no simple alternative to obtain fresh water, low-cost energy is feasible, and high standards of living allow it [3]. After World War II, the commercial exploitation of desalination focused on technologies based on thermal processes that use phase change to separate volatile solvent (water) from nonvolatile solutes (salts) [4,5]. Currently, there are two types of desalination technologies: thermal and membrane. Figure 1 shows the conventional and emerging technologies for desalination, highlighting reverse osmosis (RO) with 65% of installed capacity worldwide [1,6,7,8,9].

Nowadays, the greatest challenge for desalination processes is to lower operating and energy costs, through emerging technologies [10,11]. These emerging technologies may arise from taking advantage of externalities generated by synergies established in the search for innovation in integrated operation models [12,13]. Likewise, the incorporation of renewable energies (e.g., solar, wind, and geothermal) into desalination and integrated processes (such as RO with pressure-retarded osmosis (PRO)), has gained attention as an alternative to reduce energy costs by 50–75% of the operational costs in the conventional process [14,15,16], and 60% of the specific energy consumption (SEC) of RO [17].

In recent years, the number of publications on technoeconomic studies about desalinization processes has increased considerably. For instance, Arafat [18] related sustainability of desalination processes with technical information, concluding that current knowledge is insufficient to describe the relevance and complexity of desalination processes. Silva-Pinto and Cunha-Marques [6] evaluated the economic feasibility of different desalination and energy supply technologies, emphasizing hybrid options and the relevance of locally specific solutions. However, the authors did not illustrate their evaluation with a particular case. Proskynitopoulou and Katsoyiannis [19] reported case studies of the main desalinization technologies, highlighting energy costs and economic parameters. The authors considered desalinization as a drinking water production process applicable only in areas of water scarcity [20,21].

This article presents different desalination technologies as alternatives to conventional reverse osmosis through current state-of-the-art desalination processes considering the existing conventional and emerging technologies and a technical economic comparison between them. Further, this document offers evaluated thermal and membrane processes considering their possible implementation at a pilot plant scale (100 m^3^/d of purified water) starting from seawater at 20 °C and average salinity of 34,000 ppm.

### 1.1. Conventional and Emerging Technologies for Desalination

Desalination technologies are classified as conventional or emerging, depending on the scientific and technical development level, and their presence in the market. According to the definition proposed by Day et al. [22], emerging desalination technologies are “scientific innovations that generate incentives to make investments in the desalination process. These innovations are based on evolved technologies that improve desalination process (that is reduce energy consumption, minimize rejection and improve water quality)”. Further, a sustainability desalination industry should consider minimizing the effect of the local increase in sea salinity due to the reject stream as an important challenge.

Conventional and emerging technologies are also classified according to the type of gradient applied (pressure, electric, chemical, and thermal) and the physicochemical process involved (Table 1). Said gradient allows the separation of saline solutes from a liquid solution, through the described phenomenon.

#### 1.1.1. Conventional Thermal Technologies

**Figure 2:**
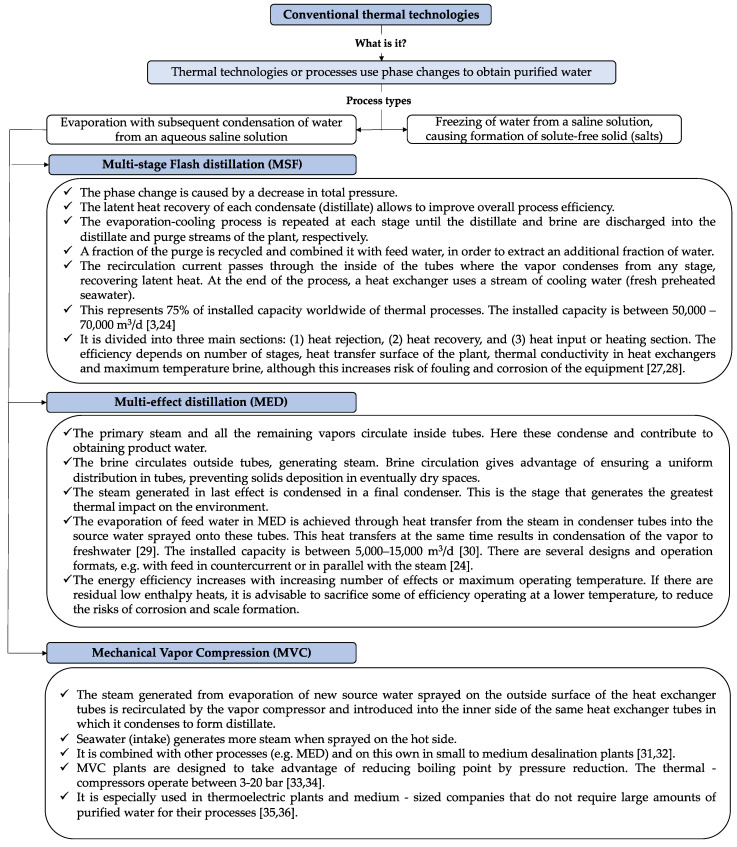
Conceptual diagram of the conventional thermal desalination technologies (Mentioned references [3,24,27,28,29,30,31,32,33,34,35,36]).

Figure 2 shows the main characteristics and types of conventional thermal desalination technologies. These processes are based on phase changes. Particularly, processes based on freezing are scarce in the market because of high investment and operation costs associated with the relevant energy demand, retention of unpleasant aromas eventually present in the feed seawater, and intensive use of refrigerants [23]. Evaporation-based processes allow obtaining a distilled, purified water, with salinity below 10 ppm. The equipment operates in stationary phase and is arranged in multiple stages in order to increase energy efficiency, profitability of the process, and avoid fouling (caused by carbonates, sulfates, silica, and other inorganic compounds) [24,25,26]. The main conventional thermal technologies are multi-stage flash distillation (MSF), multi-effect distillation (MED), and mechanical vapor compression (MVC) (see Figure 2).

The main difference between the MED and MSF processes is that while vapor is created in an MSF system through flashing, evaporation of feed water in MED is achieved through heat transfer from the steam in condenser tubes into the source water sprayed onto these tubes. This heat transfer at the same time results in vapor condensation to freshwater [29]. MVC and MED work based on similar principles. However, in MVC, the steam generated from the evaporation of new source water sprayed on the outer surface of the heat exchanger tubes is recirculated by the vapor compressor and introduced into the inner side of the same heat exchanger tubes in which it condenses to form a distillate [29]. Seawater (intake) generates more steam when sprayed on the hot side.

#### 1.1.2. Conventional Membrane Technologies

**Figure 3:**
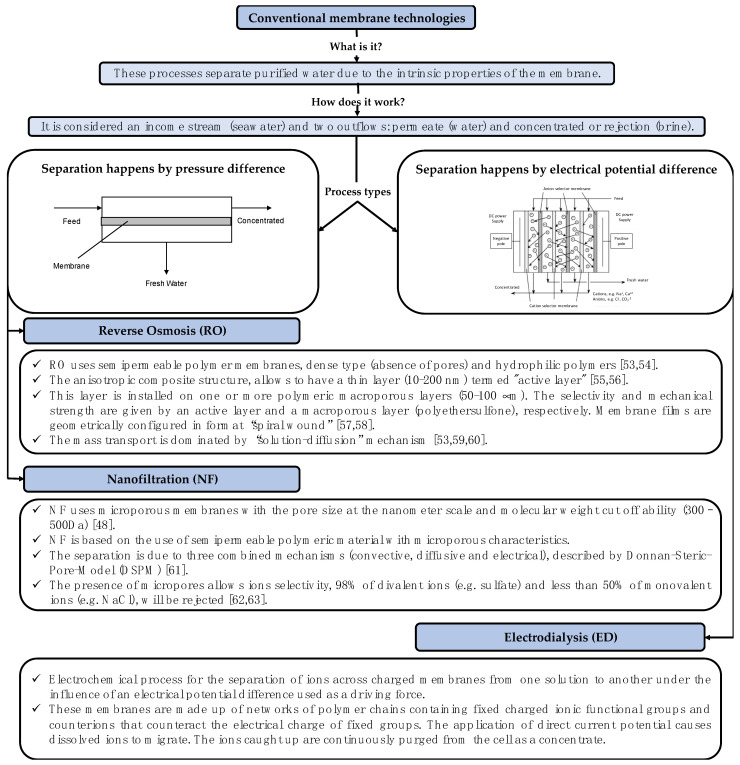
Conceptual diagram of the conventional membrane technologies applied to desalination (Mentioned references [53,54,55,56,57,58,59,60,61,62,63]).

Figure 3 shows the main characteristics and types of conventional membrane technology used for desalination. Industrial membrane processes are designed to operate continuously. However, there are transient phenomena owing to membrane fouling that force periodic cleaning routines and lead to the slow but progressive deterioration of the polymers that compose the membranes. This is an irreversible phenomenon, which ends with the replacement of the damaged membrane. For this reason, several authors investigated methods to eliminate (or avoid) fouling (and/or scaling). For example, Mangal et al. [37] investigated if antiscalants, without acid addition, can prevent calcium phosphate scaling in RO systems. The available antiscalants, tested in the study, did not provide acceptable inhibition of calcium phosphate scaling in RO applications. Landsman et al. [38] investigated the use of a hybrid electrodialysis–nanofiltration/reverse osmosis (ED-NF/RO) system to reduce fouling from calcite precipitation and calcium polysaccharide sorption to NF/RO membranes. ED pretreatment reduced calcite oversaturation and reduced flux decline during NF/RO. Low alginate concentrations (25 mg/L) limited NF/RO fouling, but high concentrations (100 mg/L) appeared to promote calcite scaling. Dhakal et al. [39] developed and demonstrated the applicability of the flow cytometry (FCM)-based bacterial growth potential (BGP) method to assess the biofouling potential in seawater (SW) RO systems using a natural microbial consortium. Sperle et al. [40] reported the potential of UVC irradiation using the recently developed UV-LEDs as an in situ pretreatment strategy for biofouling control in RO or NF systems. In contrast to UV studies carried out previously, they tested if low fluences are sufficient to not only delay the biofilm formation but further lead to a reduced hydraulic resistance of the biofilm while approaching a severe biofouling state. On the other hand, the development of ceramic membranes for membrane distillation desalination is developing, gradually replacing their polymeric counterparts due to superior properties in terms of thermal, chemical, and mechanical stabilities, as well as potentially longer service terms [41]. Bandar et al. [42] used economically and eco-friendly Saudi red clay, tetraethyl orthosilicate, ammonia, and sodium alginate powder as a binder to fabricate a ceramic membrane for membrane distillation using an extrusion technique. The prepared membrane was tested using a vacuum membrane distillation process and showed promising permeate flux and salt rejection results.

RO is the most energy-efficient technology for desalination, with much lower energy consumption than other technologies (SEC < 3.1 kWh/m^3^) [43,44,45]. Typical installed capacities fluctuate between 1000 and 600,000 m^3^/d [19,46]. External pressure required in an RO process must exceed the osmotic pressure of the aqueous feed. Since membranes offer high salt rejection levels (>99.5%), in sea water desalination operations, pressure may even double the feed osmotic pressure [47]. On the other hand, comparing nanofiltration (NF) with RO, it arises that NF technology allows partial desalination of monovalent salts such as NaCl and KCl (up to 50–60% rejection) and efficiently removes divalent ions such as sulfates and carbonates. For this reason, NF operates at lower pressures than RO due to the lower osmotic pressure gradient between the feed flow and permeate flow. Reprocessing the permeate or using consecutive desalination NF-RO stages improves the selectivity of the process. Typical NF installed capacities are found between 1000 and 100,000 m^3^/d [48,49].

Electrodialysis (ED) is a membrane-based desalination technology that operates due to an electric gradient, used for many years on brackish water (salinity < 3000 mg/L). Typical installed capacities range between 5000 and 425,000 m^3^/d [50,51,52].

#### 1.1.3. Innovations in Thermal Desalination

The main innovation in emerging desalination technologies relates to the use of renewable energy sources coupled to desalination processes. The most popular renewable energies included in these processes are solar and geothermal energy. For example, Prajapati et al. [64] investigated renewable energy sources that could be used to run desalination systems, and the potential matches between the desalination and renewable energy sources, to survey water desalination by utilizing geothermal and solar energy, to assess or identify areas which require improvement in geothermal and solar energy-driven desalination systems.

##### Solar Desalination (SD)

SD operates with direct or indirect solar energy [65]. Direct solar energy corresponds to the use of solar collectors that evaporate water and produce distillates. Indirect solar energy refers to the design of a desalination plant that uses two sub-systems: a solar collector (thermal or photovoltaic) and a desalination unit (e.g., RO).

**Figure 4:**
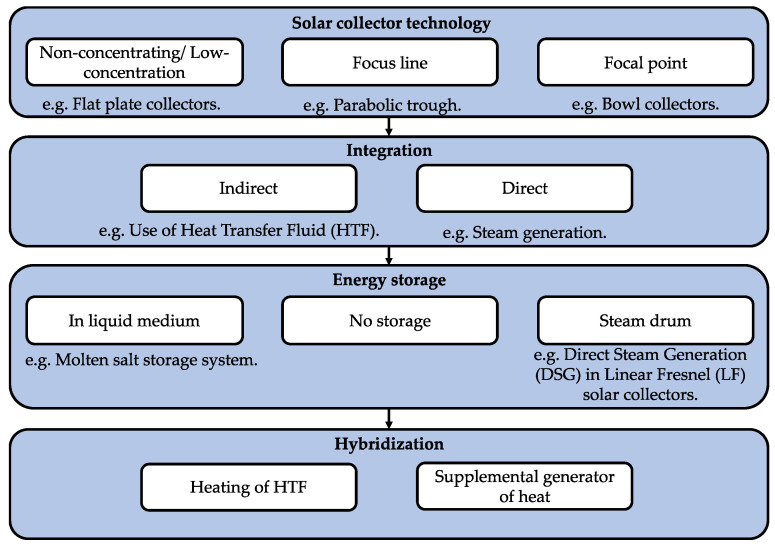
Conceptual diagram depicting the classification of solar collector technologies.

Figure 4 shows an overview of solar energy capture. Solar collector technology for thermal distillation processes depends on the maximum temperature level (Tm) in the evaporation–condensation phenomena, i.e., Tm < 130 °C, low-temperature solar energy (LTSE) and Tm > 130 °C, high-temperature solar energy (HTSE). LTSE is generated in non-concentrated or low-concentration collectors and uses simple solar fields, i.e., without moving parts and with low investment and low operating costs [66,67]. HTSEs use mirrors to focus solar irradiation from large surfaces (aperture area) onto the small surface of the receiver. This reduces hot surface areas in solar fields. On the other hand, the integration of heat transfer fluid implies that the pipes should be thermally isolated. Subsequently, desalination technologies that utilize thermal energy and thus require thermal energy storage (TES) for uninterrupted process operation are MED, MSF, low-temperature MED, humidification–dehumidification, low-temperature desalination, and membrane distillation [68]. The most commonly used TES methods are thermal oil, pressurized water, molten salts, and storage heaters. These systems have maximum operating temperatures of 395, 150, 250–550, and 550 °C, respectively [68,69,70].

**Figure 5:**
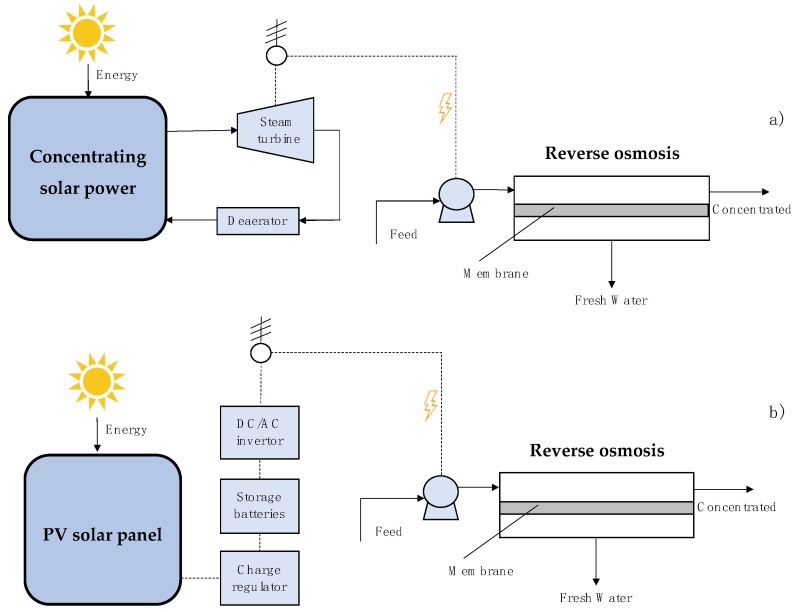
Schematic view of (**a**) Concentrating solar power + Reverse osmosis (CSP/RO) plant, (**b**) Photovoltaic solar panel + Reverse osmosis (PV/RO) plant.

Figure 5a depicts a desalination plant that uses a solar thermal energy concentration device (CSP) and RO (CSP/RO). The CSP captures thermal energy to produce superheated steam. Some CSPs allow storing heat in a sub-process based on molten salts [71,72]. Superheated steam flows to a turbine to produce electrical energy. The high-pressure pump operates on the generated electrical energy. Figure 5b represents a desalination plant that uses photovoltaic cells and RO (PV/RO). This process is useful for small desalination equipment (<0.2 L/s) and brackish water that requires less pumping power [73,74,75,76]. PV/RO requires batteries to maintain a continuous operation [77,78].

It follows that the indirect systems are more efficient and therefore are more adequate for use in industrial scale production.

##### Geothermal Desalination (GD)

Geothermal energy extraction has the advantage of being independent of the season and climate changes. Geothermal energy sources (GESs) can be applied in both membrane and thermal desalination processes, depending on the location, and on the physical and chemical characteristics of geothermal water. GD recovers heat from a GES to evaporate seawater.

**Figure 6:**
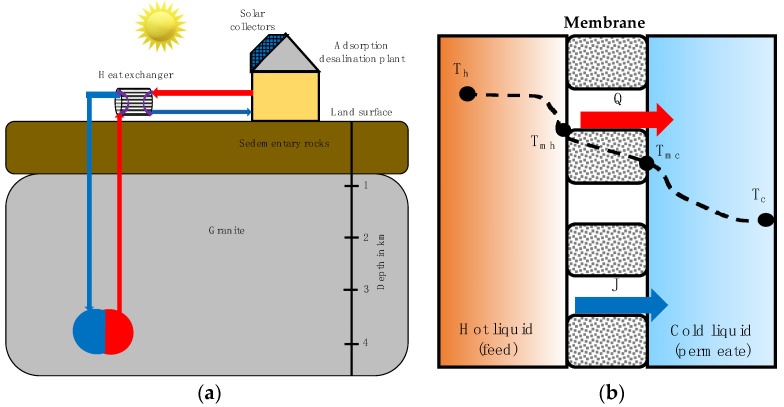
(**a**) Combined-cycle geothermal and solar-powered desalination. The system is powered by solar energy during daylight hours and by geothermal energy during nighttime and cloudy days. (**b**) Principle of direct contact membrane distillation process. Based on Ghaffour et al. [81].

Membrane processes integrated with GES are still under development (see Figure 6). Gude [79] investigated the potential of geothermal energy sources for MD. They concluded that roughly a 6.1% increase in permeate flow rate for every 2-degree temperature difference (in feed water) can be achieved by utilizing process waste heat sources. Assad et al. [80] investigated two technologies for water desalination using geothermal-powered systems that are presented and discussed. These technologies are promising, especially in the Gulf region, where geothermal energy is widely available.

#### 1.1.4. Innovations in Membrane Processes

The most relevant innovations refer to the synthesis or modification of polymeric materials that allow increasing the productivity and selectivity of desalination processes. Similarly, innovations in conventional technologies were developed, combining mass transport mechanisms, separation gradients, and renewable energy sources. In all cases, the objective is to obtain a reliable desalinated water production technology at the lowest cost.

##### Nanomembranes (NMs)

NMs are membranes that contain nanoparticles (zeolitic type or metal oxide) in the active layer of the polymer matrix, e.g., polymerized polyamide, aiming at improving hydrophilicity, productivity, and salt rejection [82].

NMs, also known as thin film nanocomposite membranes, are prepared by the phase inversion method (inorganic–organic mixed matrix). The common nanomaterial used in NM synthesis is TiO_2_ due to high chemical stability, low toxicity, defouling, and photocatalytic properties and availability [83]. The organic NM portion allows diverse geometries due to its flexibility, high density of spiral wound packing, ease of manufacturing, and good permeability and selectivity. In turn, the inorganic part of the NMs allows high surface charge density, negative zeta potential, which minimizes fouling problems, ion exchange capacity, which increases rejection, high hydrophilicity, which increases permeability, salt selectivity, and biocidal and antimicrobial capacity, which reduces bio-fouling.

**Figure 7:**
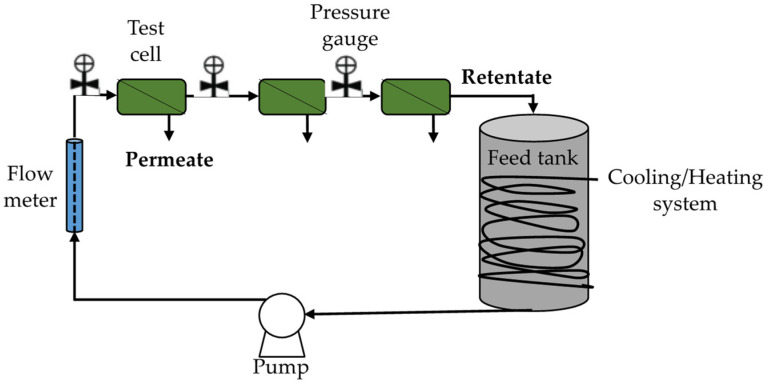
Example of scheme of a cross-flow desalination system for nanomembranes. Based on Safarpour et al. [83].

Recently, some authors compared NMs with conventional reverse osmosis membranes at the same pressure, temperature, salinity, and flow rate, resulting in higher productivity (10–20%), constant selectivity (99.5–99.8%), a fouling decrease, and lower energy demand [84,85,86] (see Figure 7).

##### Membrane Distillation (MD)

**Figure 8:**
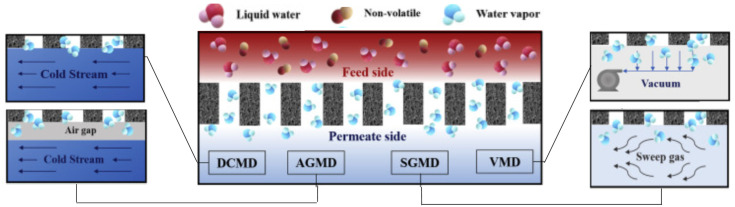
Schematic illustration of different approaches for the conventional MD process: direct contact membrane distillation (DCMD), air-gap membrane distillation (AGMD), sweep gas membrane distillation (SGMD), and vacuum membrane distillation (VMD). Based on Anvari et al. [87].

MD uses hydrophobic polymeric membranes (e.g., PP, PTFE, PVDF) of porosities between 0.01 and 0.5 μm. The vapor distillate in MD may be produced by temperature, partial pressure, or vacuum gradients. Direct contact membrane distillation (DCMD), air-gap membrane distillation (AGMD), sweep gas membrane distillation (SGMD), and vacuum membrane distillation (VMD) are different MD configurations that have reached further applications (see Figure 8).

DCMD and VMD are the most investigated MD configurations [88]. DCMD consists of two liquid phases placed in direct contact on both sides of a microporous membrane. Inside the pore, the formation of a stagnant gas phase occurs. The temperature gradient produces a superficial evaporation on one side of the membrane and a superficial condensation on the opposite side of it. Furthermore, diffusive mass transfer occurs due to the partial pressure gradient of evaporated components. Several authors reported a permeate (evaporate) flux between 5 and 30 (L/hm^2^) for thermal gradients between 20 and 40 °C [89,90,91,92,93,94]. Additionally, VMD has a productivity greater than DCMD because it allows operating at a pressure lower than 5 kPa, and in this way, obtains high evaporate fluxes [93,94,95].

Recently, Memsys Water Technologies GmbH (Schwabmünchen, Germany) combined the advantages of MED with those of MD processes, resulting in V-MEMD, composed of stages that operate under vacuum with an adequate temperature difference to increase flux through the membrane. In V-MEMD, refrigeration flux in the last stage (highest vacuum) is partly used as preheated feed to the system. The feed passes through each stage, evaporating part way in a cross-flow with the vapor that comes from the thermal system [96].

##### Forward Osmosis (FO)

This emerging technology has been addressed as a sustainable and cost-efficient solution to classical membrane-based separation technologies such as RO and membrane distillation [97].

**Figure 9:**
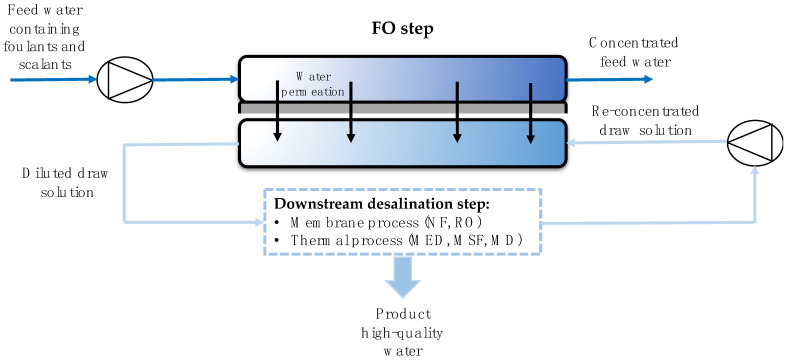
Schematic view of FO pretreatment in hybrid systems for desalination. Based on Tiraferri [102].

Water desalination by means of FO processes consists of the osmotic dilution of the draw solution (DS) and freshwater production from the diluted DS [98,99,100]. FO uses an osmotic pressure differential across the membrane, rather than a hydraulic pressure differential (as in RO), as the driving force for water transport through the membrane. Without the requirement for externally applied hydraulic pressure, FO is installed with a simple and inexpensive low-pressure apparatus, which in turn can reduce the capital costs associated with pumping and system construction [101]. Figure 9 shows FO joined to a conventional desalination process. Re-concentrated DS flux allows pure water flows from feed water. The synchronized operation of the two processes is a key parameter in the whole process design so that desalination is simple, robust, and reliable.

The FO membrane performance mainly depends on the DS properties. The ideal DS should provide high osmotic pressure (higher than the feed osmotic pressure), be inexpensive and nontoxic, provide easy recovery of the solute, be stable, and reduce internal concentration polarization [100].

Recently, FO membrane performance towards desalination was improved by modifying both the DS and FO membranes [103,104,105]. In 2012, Modern Water PLC built “Manipulated Osmosis Desalination (MOD)” in Al Najdah (Oman). MOD was the first commercial FO plant producing 200 m^3^/d [106].

##### Reverse Electrodialysis (RED) and Shock Electrodialysis (SED)

RED operates in the same way as ED except for the fact that the voltage is applied in reverse about three to four times an hour with an overall water recovery of 97% [107]. The electrode polarity is reversed at regular intervals for minimizing fouling on the membranes. Therefore, the pretreatments and membrane cleaning are minimal. Rejection of 75–90% is achieved, which depends on ion type and valence, electrical potential, and feeding speed [108]. Industrial applications of RED are brackish water desalination and, at pilot scale, seawater. To the extent that this process can be coupled with a renewable energy source, it will be possible to justify its application for higher salinity waters [109]. On the other hand, Tristan et al. [110] surveyed the life cycle assessment of salinity gradient energy capture by reverse electrodialysis (SGE-RED). They quantified (i) the environmental loads per 1.0 kWh generated by a standalone RED unit and then (ii) the environmental burdens related to the energy provision from an up-scaled RED system to a seawater RO desalination plant per 1.0 m^3^ of desalted water. The RED unit’s assessment results show that SGE-RED is environmentally competitive with other renewable sources such as photovoltaics or wind. The high salinity solution treated with RED has a lower salt concentration and serves as feed solution to the RO unit to reduce the pump work.

SED is a developing technology that purifies water using polarization zones by the concentration of ions in porous media, adjacent to an ion-selective membrane. A SED cell consists of two ion exchange membranes or electrodes between which feedwater flows through a charged porous medium with thin double layers that act as a “leaky membrane” [111]. When a current passes through the SED cell, an ion-depleted zone is formed along an ion-selective element (the cathode). As the applied voltage is increased, ion concentration near this element approaches zero, and the system can reach the classical diffusion-limited current [106]. Actually, SED operates at small scales, so it holds promise as a decentralized, point-of-use desalination system. SED could be incorporated as a predesalination stage in the RO process, increasing water recovery, decreasing energy consumption, and providing an affordable cost. Alkhadra et al. [112] removed from 70 to 99% of ions of artificial seawater (37,685 ppm) using SED.

#### 1.1.5. Emerging Membrane Processes

Two processes based on polymeric porous media are currently in development and deserve to be considered: graphene oxide membranes (GMs) and aquaporin-based biomimetic membranes (ABMs). On the other hand, hybrid and integrated systems (FO-MD, RO-MD, RO-PRO, etc.) are considered emerging.

##### Graphene Oxide Membrane (GM)

Graphene oxide (GO), among various forms of nanomaterials, provides tremendous opportunities for rational design and tailoring for solar evaporation and film filtration because of its high absorption, porous structure, high chemical stability, hydrophilicity, and excellent anti-fouling properties [113,114]. To date, GO has been used as an absorber in solar desalination and as a filtration film in membrane desalination [115,116,117,118]. In GMs, there is usually a trade-off between salt rejection and water flux. However, this trade-off can be broken using intercalation, changing the deposition method of GO film or utilizing electrostatic interaction between GO and ions. Currently, GMs are being studied on a laboratory scale [119]. Freire and Pacheco [120] determined that the energy consumption of GMs is less than that of a commercial membrane, since they required lower operating pressures and the coefficient of water mass transport was higher, obtaining permeates with total dissolved solids lower than and equal to 500 ppm.

##### Aquaporin-Based Biomimetic Membrane (ABM)

Aquaporins (AQPs) are pore-forming proteins in biological cells. These are composed of a bundle of six transmembrane α-helices embedded in the cell membrane. The amino and carboxyl ends face the inside of the cell, whereas the halves resemble each other, apparently repeating a pattern of nucleotides [121,122,123]. Under the right conditions, AQP forms a water channel that selectively transports the water molecules across while excluding ionic species or other polar molecules. This novel property makes AQP a perfect model for the formulation of a low-energy water purification system in seawater desalination [124]. Amy et al. [7] reported that ABMs are being developed as ultrahigh permeability (UHP) RO membranes; with impregnation of AQP (or vesicles) into a polymeric matrix, AQP can provide water channeling/gating, leading to controlled water permeability and ion selectivity.

The major obstacle for ABMs is the scaling up for industry applications, since only small-area membranes have been synthesized due to the highly specialized synthesis techniques. The ABM developed by Zhao et al. [125] had good mechanical stability for periods of weeks to months with stable flux and rejection. Additionally, its permeability was ~40% higher than commercial brackish water RO membrane (BW30) and an order of magnitude higher than seawater RO membrane (SW30HR), which clearly demonstrated the great potential of ABMs for desalination application [124]. ABMs have the potential to reduce energy costs for water treatments [126]. However, these membranes are still at the bench scale and more advancements are necessary to improve their chemical resistance and mechanical strength [127].

##### Hybrid and Integrated Systems

In recent years, hybrid membrane processes have allowed the achievement of better indicators of the desalination process. For example, Ghaffour et al. [128] presents the state of the art of MD hybrids with different separation processes including RO, PRO, FO, MVC, electrocoagulation, ED, MSF, MED, crystallization, and adsorption with a focus on water production and energy efficiency enhancement. Each of these processes has advantages at the cost of more or less severe drawbacks and their association with MD offers improvement opportunities. Kim et al. [129] proposed a novel module design to integrate FO and MD. The two processes are sealed in one module and operated simultaneously, making the system compact and suitable for a wide range of applications. Results indicated that initial draw solution (MD feed) flow rate and concentration are the most important factors for stable operation of the integrated module.

#### 1.1.6. Technological Improvements Based on Energy Recuperation

Arafat [18] defined two categories for brine energy-recovery systems: the use of pressure exchangers for the direct transfer of the brine pressure to the feed flow, and the use of turbines and pumps that transform rejection pressure energy into mechanical power. Currently, RO plants with a production greater than 3000 m^3^/d incorporate energy recovery devices, e.g., Pelton turbine, turbocharger, and pressure exchanger (PX device). These devices recover energy from the same desalination process.

**Figure 10:**
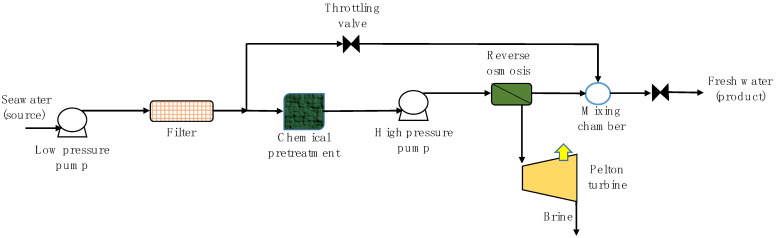
Schematic diagram of SWRO desalination plant with energy-recovery turbine. Based on El-Emam & Dincer [132].

The Pelton turbine transforms the rejection pressure into kinetic energy (see Figure 10). The pressurized liquid hits a wheel with vanes that is attached to a high-pressure pump motor. The turbocharger is a compact energy recovery unit, in which a pump and turbine are connected inversely and are provided with a single shaft. The turbine of the turbocharger converts hydraulic pressure energy into mechanical energy that can be used by the pump, allowing an increase in fluid pressure. The PX device directly transfers the high pressure of reject brine to seawater, without previously converting it into mechanical rotation energy. The system uses the principle of positive displacement and isobaric chambers. In fact, the energy savings achieved through these three systems can reach 40%, working with high efficiency (up to 97%) [130]. This represents an SEC close to 2.5 kWh/m^3^ [131], i.e., according to SEC values reported by Chandwankar and Nowak [24], an SEC equal to that of the MED process and 30% less than the MSF process.

## 2. Materials and Methods

The comparison method applied in this research can be used in any geographical context. In this case, the expert panel method was applied to determine the best option (technical and economic) of a desalination process for the current Chilean context.

Thermal and membrane processes were evaluated, aiming at applying them to a pilot scale plant that delivers 100 m^3^/d of purified water starting from seawater at 20 °C and salinity of 34,500 ppm. The desalination plant is supposed to be installed in the coastal zone of the Atacama Region (northern Chile) because:

- There is abundant solar radiation [133,134] to justify an off-grid installation using photovoltaic panels, or a hybrid arrangement with photovoltaic panels plus accumulation of electrical energy in batteries or with partial supply of electrical energy from the network. Osorio-Aravena et al. [135] reported that, in Chile, renewable electricity will mainly come from solar PV and wind energy technologies. Solar PV and wind energy installed capacities across all sectors would increase from 1.1 GW and 0.8 GW in 2015 to 43.6 GW and 24.8 GW by 2050, respectively. As a consequence, the levelized cost of energy will be reduced by about 25%.
- Alvez et al. [20] reported that, in Chile, large volumes of water are used in water-scarce regions where mining takes place, alongside agriculture and small communities. This situation has driven a debate around policies to increase the use of seawater to satisfy the water demand of the mining industry.
- Fragkou and Budds [136] argued that, in Chile, desalination serves to disarticulate drinking water from fresh water, with implications for economic growth, social development, and water policy. They show that desalination entails more than providing additional water to alleviate shortages, and rather constitutes a strategy that permits the reorganization of water sources so as to allow new forms of capital accumulation, through both the water industry as well as the major industries that are threatened by scarcity. They argue that this has three important implications: (1) replacing freshwater with desalinated water for human consumption changes the social relations of control over water, by rendering consumers dependent on desalination plants and their risks, (2) this disarticulation serves to liberate fresh water to sustain the same industries that encroached on drinking water sources, and (3) as a supply-led solution, desalination alleviates some of the water shortages that had been attributed to Chile’s water market model, thereby reducing pressure for reform.
- Spenceley [137] informed that, in northern Chile, technologically advanced desalination plants are built along the coast, and the desalinated water is moved through an accompanying conveyance system—a complex system of pipelines and pumping infrastructure—over long distances. The resulting brine is released back into the sea through a sophisticated dispersion system designed to reduce brine concentrations to ambient levels efficiently and over the shortest distances possible. Herrera-Leon et al. [138] identified that eleven desalination plants at the industrial scale are operating in Chile (until 2018), producing 5868 l/s of desalinated water. Additionally, there are ten desalination projects in different stages of evaluation, which will increase the desalination capacity by 116.5% to reach a total of 12,706 l/s in the coming years.
- Due to the high energy demand of desalination techniques, there is a great need for alternatives to reduce the salinity from seawater [139]. In addition, secondary ions, such as calcium and magnesium, in the SW cause scale problems in reverse osmosis plants, mining, and others industries, such as cooling systems. These problems cause increased costs and reduce the efficiency of these processes [140].
- The use of brackish groundwater often brings risks and obligations to an agricultural system. The application of desalinated water for irrigation can promote soil hydrological functions [141]. However, the disposal of RO concentrate from an inland desalination system can be problematic, and its sustainable management is a major environmental challenge that restricts the widespread application of RO for groundwater desalination [142].

Initially, the classification of information and definition of the technologies to be compared were based on a systematic literature review. Subsequently, the comparative analysis was divided into five categories:

(1) Technical characteristics,
(2) Scale-up potential,
(3) Temperature effect,
(4) Electrical supply options,
(5) Economic viability.

Each category was discussed through an expert panel method [143,144,145], composed of five professionals with ample experience in desalination processes. The participants were selected on the basis of professional excellence, landmark publications, and significant teaching experience about desalination processes in Chile. The expert panel analyzed the information from the systematic review and a score was agreed upon according to the criteria shown in Table 2 and Table 3.

Table 2 shows the four criteria levels applied to technical characteristics (category 1): technological development level, operation mode, feasibility of operation with Non-conventional renewable energies (NCRE), pretreatment level, and ease of industrial scaling. The difference in score (1-4 and 1-3) is due to the fact that the expert panel decided to give higher relevance to “technological development level” and “operation mode”. The criteria mean:

- *Incipient:* technological development at theoretical and/or laboratory scale.
- *Emerging:* technological development prototype at pilot scale.
- *Medium–high:* technological development as commercial-scale equipment.
- *High:* consolidated technological development and conventional technology, in continuous improvement.
- *Complex:* unstable operation.
- Moderately complex: stable operation.
- *Relatively easy:* complicated operation and operation with automation.
- *Easy:* very stable process operation and of easy automation.
- *low:* the current context does not allow operation with NCRE.
- *medium:* the future context does not allow operation with NCRE.
- *high:* the current context allows operation with NCRE.
- *demanding:* the pretreatment is very necessary to take care of the principal process.
- *moderate:* a complex pretreatment is necessary to achieve the goal of the principal process.
- *Simple:* a simple pretreatment is necessary to achieve the goal of the principal process.
- low*: the current context does not allow industrial scaling.
- medium*: the future context does not allow industrial scaling.
- high*: the current context allows industrial scaling.

The results of this first evaluation allowed us to determine the technologies with the best characteristics to be applied in Chile and thus focus the analysis of the following categories on those.

Four alternatives were analyzed in categories 2 and 3, based on the results of category 1:

Alternative 1 (A1): Nanofiltration;
Alternative 2 (A2): Nanomembranes;
Alternative 3 (A3): Forward Osmosis + Reverse Osmosis;
Alternative 4 (A4): Solar Distillation.

In this research, a comparison between the different alternatives was made considering a production level of 100 m^3^/d of permeate, with continuous operation for 24 h per day. Table 3 shows the parameters and criteria for the comparative analysis of the industrial scale-up potential (category 2) for the four alternatives. The criteria mean:

- *Low quality:* salt content reduced by 50%.
- *Medium quality:* salt content reduced by 51–75%.
- *High quality:* drinking water quality.
- *Worst:* productivity, quality, and cost are lower than RO.
- *Equal:* productivity, quality, and cost are similar to RO.
- *Best:* productivity, quality, and cost are higher than RO.
- Worst*: innovation is not better than the current condition.
- Equal*: innovation equals current condition.
- Best*: innovation improves the current condition.
- *None:* the integration of renewable energy into the desalination process is not possible.
- *Partial:* the integration of renewable energy into the desalination process is partially possible.
- *Yes:* the integration of renewable energy into the desalination process is possible.

For water quality according to regulations, chloride content is a critical parameter and, if water quality produced is lower than expected, then additional treatment is necessary. The innovations in the operation are the introduction of a new technology to satisfy some need of the desalination process, with science being knowledge, and technology its practice, e.g., seawater feed at a higher temperature.

The temperature effect (category 3) was analyzed for thermal and membrane desalination. Membrane desalination was analyzed based on permeate flux density considering the design equations based on the well-known solution diffusion model.

The alternative that obtained the best results in categories 1, 2, and 3 was analyzed in category 4 (electrical supply options). The analyzed options were:

(O1) photovoltaic solar plant + electrical network,
(O2) photovoltaic solar plant + batteries,
(O3) wind turbine + electrical network.

These options were established to ensure an integrated operational system, constant electricity supply, and utilization of NCRE. The electrical supply options were analyzed based on costs associated with a membrane desalination plant that produces 100 m^3^/d of permeate and consumes 2.09 kWh/m^3^ (from pretreatment to posttreatment). This plant must have a daily consumption of 188 kWh, i.e., 7.8 kW/h during the 24 h. The results of the present value of cost (PVC)/m^3^ of water were analyzed considering an internal return rate of 8% and a 20-year horizon.

Finally, analysis of category 5 (economic viability) applies to the best result obtained in category 4. The economic viability (Capital expenditure (CAPEX) and Operating expenses (OPEX)) of the electrical supply option with the lower associated costs was analyzed. We considered a 20-year time horizon. Costs associated with adduction systems, water distribution to customers, and discharge of rejection into the sea were not considered. CAPEX was determined based on the quotations provided by current suppliers in the market.

## 3. Results and Discussions

### 3.1. Comparison of Technologies (Category 1)

Table 4 and Table 5 show a summary of the technical and economic characteristics of desalination processes. This information arose from a systematic literature review. The data indicate that the emerging technology SD has an investment range equal or less than NF, negligible electricity consumption, and a low negative environmental effect. The SD shows the disadvantage of having a very high area-to-volume ratio. Currently, the active layer of NMs has been modified, thus increasing their productivity and selectivity [146,147]. They offer the best technical and economic indicators. The incorporation of aquaporins as an emerging process is the emerging lower-cost technology with the greatest potential for seawater desalination.

Nowadays, MVC, MED, and MSF contribute 40% of desalinated water worldwide (39 Mton/d) [1]. Current trends in RO are energy recovery from concentrated streams and a better membrane permeability, whereas for NF, studies aim at improving the separation efficiency to keep the energy consumption low [59]. On the other hand, FO operates in close cycles with other desalination technologies, thus reducing energy consumption. Finally, GO and ABMs are in the early phases of research, with the expectation that they will achieve their technological maturity and therefore become commercially viable over a 10-year horizon.

The maintenance cost is similar for all technologies, while the technologies that use lower streams presented lower investment and operating costs. This behavior is in agreement with that reported in the literature [88,100].

All desalination technologies have the drawback of returning a concentrated solution to the sea; therefore, the minimization of the effect of a local sea salinity increase due to the reject flow is now the technological challenge.

Table 6 shows the evaluation of the technical characteristics performed by the expert panel. The technologies inserted in the market comply with more than 70% of the technological characteristics. The data suggest that NF and NM have the highest score (after RO), i.e., these technologies have favorable characteristics to stay in the membrane market. This behavior agrees with that reported in the literature [49,85]. RO does not have maximum score due to its demanding level of pretreatment.

Based on the previous results, the technologies chosen by the expert panel to be studied in detail were: NF, NM, FO + RO, and SD. This is because: (1) the technologies supplied with fossil fuel as an energy source were discarded, given the energy context in Northern Chile, (2) SD is an emerging technology with great potential for development in Northern Chile due to greater solar radiation in the area, (3) RO, NF, and NM were the membrane processes that obtained the highest scores, and (4) RO + FO is an emerging process that allows reducing the SEC of traditional RO.

### 3.2. Industrial Scale-Up Potential (Category 2)

Table 7 shows the evaluation criteria to define the industrial scale-up potential of the four alternatives: NF, NM, FO+RO, and SD. NM achieved the highest score. This evaluation considers that NF increases its productivity by increasing water temperature, but without modifying the saline content of the product. This forces a reprocessing of the permeate at several stages, increasing production cost. Instead, SD achieves high-quality water and productivity, but costs are higher than RO. Finally, NM presents good prospects for industrial scale-up. This technology uses electricity as an energy source; therefore, it would be possible to incorporate renewable energy into its operation. NM is competitive against traditional RO. This behavior agrees with that reported in the literature [146,147,148].

### 3.3. Temperature Effect on Membrane Productivity (Category 3)

Temperature is a variable that influences desalination processes. The SD process, based on phase change, will increase its productivity (evaporation flow) by less than 5% if the operating temperature increases from 20 to 30 °C. In contrast, NM and RO processes, based on the mass transport through semi-permeable dense polymers, will increase their productivity by around 20%, consistent with what the solution diffusion model predicts [149,150].

### 3.4. Electrical Supply Options (Category 4)

The preceding results indicate that the best alternative is NM. O1 and O2 consider the use of solar panels installed near the plant. The photovoltaic solar plant loses 20% of its energy, therefore, 9.8 kWh for 24 h are required. These energy requirements are met all year round for 7 h (10 a.m.–5 p.m.) due to solar radiation in Northern Chile. Instead, O3 considers a 25 kW wind turbine. This equipment delivers the energy requirement to the plant for 5 to 7 h (September to April). The conversion efficiency from wind energy to electrical energy is 25–45%.

Table 8 shows the CAPEX calculated for each option (O1, O2, and O3), where O1 presents the lowest CAPEX (equipment).

These results suggest that O1 is the most favorable in the coastal zone of Northern Chile, due to the great potential of solar energy and low investment and maintenance costs.

### 3.5. Economic Viability (Category 5)

All three options have the seawater desalination system in common, therefore the options share the same economic analysis. Table 9 shows the CAPEX of the seawater desalination plant installation and operation.

For the comparative analysis of the three options, the PVC was used, considering: internal rate of return (IRR) equal to 8%, total evaluation period of 20 years, and operating costs, such as: labor, chemicals, clean-in-place (CIP) cleaning, membrane replacement, spare parts, and plant maintenance, resulting in 0.39 USD/m^3^ of purified water.

The relevant difference between the options is the cost associated with the type of electrical energy. In this way, the PVC was calculated considering exclusively the item energy, both in CAPEX and OPEX. This implies that O1, O2, and O3 have a PVC of USD 0.085/m^3^, USD 0.15/m^3^, and USD 0.115/m^3^, respectively. In addition, if the plant operates only with an electrical network, then its PVC is USD 0.42/m^3^.

The results indicate that the use of NM together with a mixed energy supply system (Alternative 2 + Option 1) is profitable. The cost of a seawater desalination system with a daily production of 100 m^3^/d, and a 40 ft container with thermal and acoustic insulation, is 71–74% of the overall cost of the project. When incorporating the cost of a seawater desalination system with a daily production of 100 m^3^/d and total operating costs, in this case, a PVC of USD 0.81/m^3^ is obtained, that is, between 32% and 35% lower compared to the conventional reverse osmosis system, for a plant with a capacity of 100 m^3^/d.

These results reinforce similar studies by some authors in other geographic locations. For example, Mollahosseini et al. [151] analyzed Iran’s general water background and its renewable energy status, in addition to the potential in renewable energy-assisted desalination (RED). The research suggests that Iran’s potential in RED water production is more than 28 billion m^3^ in the case where only wind and solar potentials are put into practice. Thus, Iran becomes a prototype in the solutions for water scarcity in cases of proper investment and planning. Jimenez [152] evaluated the feasibility of implementing a desalination plant powered by photovoltaic solar energy in the Colombian Guajira Region. This author determined that the use of renewable energy coupled to the reverse osmosis system is the process that best adjusts to the climatic conditions of the area. Finally, Villagran [153] studied the technical–economic feasibility of the reverse osmosis process with the support of renewable wind and solar photovoltaic energy for a town in Northern Chile. This author determined investments for said plant proportional to an SEC of 2.77, 2.89, and 3.06 kWh/m^3^.

## 4. Future Research

In this research, an innovative operational arrangement was identified within the different seawater desalination technologies that can compete in regard to costs (industrial scale) with conventional RO, through a feasibility evaluation of a pilot plant located in Northern Chile. Therefore, the future implementation of the proposed pilot plant will allow obtaining the experimental data.

## 5. Conclusions

Currently, membrane desalination processes that use energy recuperators have an SEC 50% lower than thermal desalination processes. NM technology exhibits the most favorable technical characteristics and the best economic indexes to consolidate in the desalination market. Even further, NM represents a competitive alternative versus conventional RO process. A profitable desalination process must consider NM, use of energy recuperators, and mixed energy supply. NM + photovoltaic solar plant + electrical network is the most favorable option in the coastal zone of Northern Chile, due to the great potential of solar energy and low investment and maintenance costs. The incorporation of aquaporins as an emerging process is the emerging lower-cost technology with the greatest potential for seawater desalination. A challenge for all desalination technologies is to minimize the effect of local sea salinity increases due to reject streams. This document presents an up-to-date overview of the impact of emerging technologies on desalinated water quality, process costs, productivity, renewable energy use, and separation efficiency. Said information shows that new desalination technologies are more efficient and competitive. NMs are probably the technology that will be used in the future for desalination processes.

## Figures and Tables

**Table 1:** Summary of conventional and emerging technologies for desalination according to physicochemical process and external gradient.

	Technology	External Gradient	Physicochemical Process
**Conventional**	Multi-stage flash distillation (MSF)	Thermal	Phase change
	Multi-effect distillation (MED)		
	Mechanical vapor compression (MVC)		
	Freezing		
	Reverse osmosis (RO)	Total pressure	Physical,
	Nanofiltration (NF)		without phase change
	Electrodialysis (ED)	Electric	Electric,
			without phase change
	Ion exchange (IE)	Chemical	Chemical,
			without phase change
**Emerging**	Solar distillation (SD)	Thermal	Phase change
	Solar thermal with RO		
	Solar photovoltaic with RO		
	Geothermal desalination		
	Nanomembranes (NMs)	Chemical	Physical, membrane properties improved
	Membrane distillation (MD)	Thermal	Phase change
	Forward osmosis (FO)	Osmotic pressure	Chemical,
			with phase change
	Reverse electrodialysis (RED)	Electric	Electric
	Shock electrodialysis (SED)		without phase change
	Biomimetics (aquaporins)	Chemical	Chemical
	Graphene membrane (GM)		

**Table 2:** Criteria to analyze the most relevant characteristics of conventional and emerging technologies.

**Characteristic**	**Criteria**			
	**1**	**2**	**3**	**4**
Technological Development Level	*Incipient*	*Emerging*	*Medium–high*	*High*
Operation Mode	*Complex*	*Moderately complex*	*Relatively easy*	*Easy*
**Characteristic**	**1**	**2**	**3**	
Feasibility of Operation with NCRE	*low*	*medium*	*high*	
Pretreatment Level	*demanding*	*moderate*	*simple*	
Ease of Industrial Scaling	Low*	Medium*	High*	

**Table 3:** Criteria to analyze the industrial scale-up potential (category 2).

Parameter	Criteria		
	0	0.5	1
Water quality according to regulations	*Low quality*	*Medium quality*	*High quality*
Productivity, quality, and cost vs. RO	*Worst*	*Equal*	*Best*
Innovations in the operation	Worst*	Equal*	Best*
Integration of renewable energy	*None*	*Partial*	*Yes*

**Table 4:** Summary of desalination technology characteristics (part 1).

Technology	Process Type	Separation Gradient	Principal Equipment	Separation Mechanism	Energy Source	Investment (USD/m^3^ d)	Electricity Consumption (kWh/m^3^)
MVC	Thermal/Traditional	Temperature	Thermal compressors	Liquid–vapor equilibrium	Fossil fuel	1000–1200	5.5–6.5
MED	Thermal/Traditional	Temperature	Heat exchanger (multi-effect)	Liquid–vapor equilibrium	Fossil fuel	850–2000	1.5–2.5
MSF	Thermal/Traditional	Temperature	Heat exchanger (flash)	Liquid–vapor equilibrium	Fossil fuel	900–2000	3.0–4.5
SD	Thermal/Emerging	Temperature	Solar collectors	Liquid–vapor equilibrium	Solar radiation	500–1000	0.05
RO	Membrane/Traditional	Total pressure	Dense membrane	Selective permeation. Solution diffusion model	Electricity	800–2500	2.5–3.5
NF	Membrane/Traditional	Total pressure	Microporous membrane	Ions size vs. pore size Donnan steric partition pore model (DSPM)	Electricity	600–1000	1.0–2.0
NM	Membrane/Modified	Total pressure	Dense and modified membrane	Selective permeation improved with nanoparticles Solution diffusion model, share adsorption–desorption	Electricity	800–2000	2.0–3.0
MD	Membrane/Emerging	Partial pressure and temperature	Mesoporous–Macroporous membrane	Liquid–vapor equilibrium	Electricity	<800	0.1 (DCMD) 1.0 (VMD)
FO	Membrane/Emerging	Concentration	Dense membrane	Osmotic pressure	Electricity	<800	0.15 for each recirculation
GO	Membrane/Emerging	Total pressure	Monolayer of graphite atoms linked by covalent bond	Molecular sieve + adsorption–desorption	Electricity, chemical	*	*
ABM	Biomembrane/Emerging	Concentration	Aquaporin water channels in membrane	Hydrogen bonds with cell membranes	Electricity, chemical	*	*

* The incipient development of these technologies does not allow reporting information at the industrial level.

**Table 5:** Summary of desalination technology characteristics (part 2).

Technology	Indication with Regard to Energy Consumption	Operation Cost (USD/m^3^)	Maintenance Cost (USD/m^3^)	Desalinated Water Quality (ppm)	Area/Volume	Effect on Environment
MVC	a low enthalpy heat source allows a competitive process	0.5–5.0	0.10	10	medium	CO_2_ emissions, local sea temperature increase
MED	a low enthalpy heat source allows a competitive process	0.4–5.0	0.15	10	medium	CO_2_ emissions, local sea temperature increase
MSF	a low enthalpy heat source allows a competitive process	0.4–5.0	0.15	10	medium	CO_2_ emissions, local sea temperature increase
SD	operation with minimal energy consumption	0.05–0.20	0.05–0.10	10	high	low effects
RO	100% dependent on electrical supply	0.8–2.0	0.10	300–500	low	local sea salinity increase
NF	100% dependent on electrical supply	0.25–0.5	0.10	15,000 (1 stage)	low	local sea salinity increase
NM	100% dependent on electrical supply	0.6–1.8	0.10	200–350	low	local sea salinity increase
MD	operation with low energy consumption	1.2–2.5	0.10	<10	high	local sea salinity minimal increase
FO	operation with low energy consumption	0.3	0.10	<10	medium	local sea salinity minimal increase
GO	operation with low energy consumption	*	*	<200	low	local sea salinity increase
ABM	operation with low energy consumption	*	*	<200	low	local sea salinity increase

* The incipient development of these technologies does not allow reporting information at the industrial level.

**Table 6:** Results of the comparative evaluation of desalination technology characteristics.

Technology	Technological Development Level	Operation Mode	Feasibility of Operation with NCRE	Pretreatment Level	Ease of Industrial Scaling	Total	Compliance Percentage (%)
MVC	4	4	1	2	1	12	70.6
MED	4	4	2	2	2	14	82.4
MSF	4	4	2	2	2	14	82.4
SD	3	3	3	2	2	13	76.5
RO	4	4	3	1	3	15	88.2
NF	3	4	3	1	3	14	82.4
NM	3	4	3	1	3	14	82.4
MD	1	2	2	2	1	8	47.1
FO	3	3	2	2	2	12	70.6
GO	1	1	3	1	1	7	41.2
ABM	1	1	3	1	1	7	41.2

**Table 7:** Results of the comparative evaluation of the industrial scale-up potential for NF, NM, FO + RO, and SD.

Parameter	Technology			
	NF	NM	FO + RO	SD
Water Quality According to Regulations	0	1	1	1
Productivity, Quality, and Cost vs. RO	0	1	0.5	0.5
Innovations in the Operation	0.5	1	1	0.5
Integration of Renewable Energy	1	1	1	1
Total Compliance Percentage (%)	1.5	4	3.5	3
	37.5	100	87.5	75

**Table 8:** Capital expenditure (equipment) for O1, O2, and O3.

Option	Item	Quantity	Unit	USD
O1	Solar panel (310 W)	80	un	15,856
	Inverter	4	un	4865
	Other components			1719
	Total (solar park)			22,440
O2	Solar panel (310 W)	80	un	25,369
	Inverter	4	un	4865
	Storage batteries			38,400
	Other components			3093
	Total (solar park)			65,541
O3	Wind turbine (25 kW)	25	un	45,688
	Total			45,688

**Table 9:** Summary of CAPEX of the seawater desalination system.

Activity	Cost (USD)
Seawater desalination system with daily production of 100 m^3^/d, and a 40 ft container with thermal and acoustic insulation	196,442
Potabilization system	12,522
Supervision and assembly	32,496
**Total**	**241,460**

## Data Availability

All data will be available from the corresponding author on reasonable request.
